# Supplementary material for: Transcriptomic Insights into Mechanisms of Early Seed Maturation in the Garden Pea (Pisum sativum L.)
Source: Cells. 2020 Mar 23;9(3):779. doi: 10.3390/cells9030779 (PMC7140803; doi:10.3390/cells9030779)

# Zhongwan−6,25(1)

## Global Summary

%DE = 0.29

# genes with fdr < 0.2 = 181 ( 80 + / 101 −)

# genes with fdr < 0.1 = 166 ( 75 + / 91 −)

# genes with fdr < 0.05 = 146 ( 63 + / 83 −)

# genes with fdr < 0.01 = 137 ( 57 + / 80 −)

# genes in genesets = 0

<FC> = 0

<t−score> = 0

<p−value> = 0

<fdr> = 0.71

**Portrait**

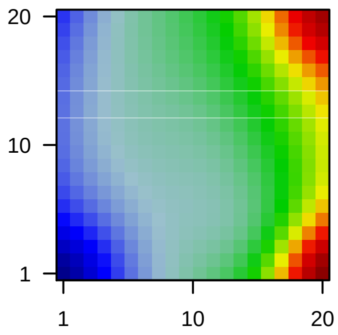


**Top 100 DE genes**

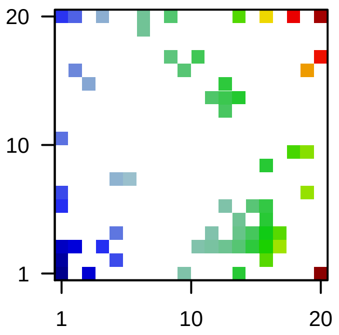


## Global Genelist

| Rank | ID | log(FC) | p−value | fdr | Metagene | Description |
|---|---|---|---|---|---|---|
| 1 | TRINITY_DN2 | −0.17 | 2e−16 | 1e−15 | 14 x 3 | |
| 2 | TRINITY_DN1 | 5.71 | 2e−16 | 1e−15 | 20 x 1 | |
| 3 | TRINITY_DN1 | 3.01 | 2e−16 | 1e−15 | 20 x 20 | |
| 4 | TRINITY_DN5 | 0.7 | 2e−16 | 1e−15 | 2 x 20 | |
| 5 | TRINITY_DN5 | 0.27 | 2e−16 | 1e−15 | 13 x 14 | |
| 6 | TRINITY_DN1 | −0.14 | 2e−16 | 1e−15 | 10 x 1 | |
| 7 | TRINITY_DN5 | 0.22 | 2e−16 | 1e−15 | 1 x 11 | |
| 8 | TRINITY_DN2 | 2.73 | 2e−16 | 1e−15 | 20 x 1 | |
| 9 | TRINITY_DN2 | 1.3 | 2e−16 | 1e−15 | 20 x 1 | |
| 10 | TRINITY_DN3 | −0.28 | 2e−16 | 1e−15 | 1 x 1 | |
| 11 | TRINITY_DN3 | 0.19 | 2e−16 | 1e−15 | 20 x 17 | |
| 12 | TRINITY_DN3 | 0.13 | 2e−16 | 1e−15 | 19 x 10 | |
| 13 | TRINITY_DN5 | −0.15 | 2e−16 | 1e−15 | 14 x 4 | |
| 14 | TRINITY_DN4 | −0.47 | 2e−16 | 1e−15 | 15 x 3 | |
| 15 | TRINITY_DN4 | −1.13 | 2e−16 | 1e−15 | 1 x 1 | |
| 16 | TRINITY_DN4 | 1.68 | 2e−16 | 1e−15 | 18 x 20 | |
| 17 | TRINITY_DN1 | −0.17 | 2e−16 | 1e−15 | 1 x 1 | |
| 18 | TRINITY_DN3 | 0.24 | 2e−16 | 1e−15 | 20 x 1 | |
| 19 | TRINITY_DN2 | −0.17 | 2e−16 | 1e−15 | 1 x 2 | |
| 20 | TRINITY_DN2 | −0.24 | 2e−16 | 1e−15 | 1 x 1 | |

**p−values**

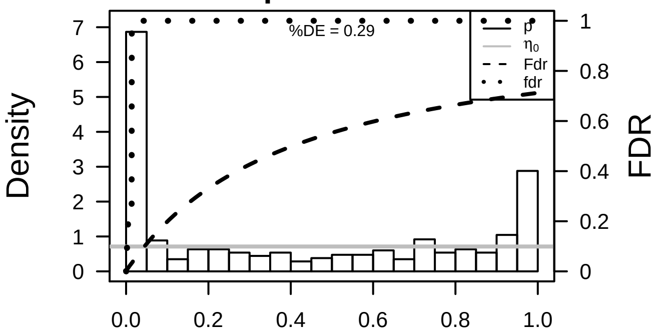

Supplement: Supplementary file 1 [file cells-09-00779-s001.zip › Supplementary materials/FileS3.zip!/FileS2/Hormones - Results/Summary Sheets - Samples/Zhongwan.6.25.1..pdf]
